# Endogenous and Therapeutic Estrogens: Maestro Conductors of the Microenvironment of ER+ Breast Cancers

**DOI:** 10.3390/cancers13153725

**Published:** 2021-07-24

**Authors:** Linda A. Schuler, Fern E. Murdoch

**Affiliations:** Department of Comparative Biosciences, University of Wisconsin-Madison, Madison, WI 53706, USA; femurdoch@wisc.edu

**Keywords:** ER+ breast cancer, tumor microenvironments, cancer immunotherapy

## Abstract

**Simple Summary:**

Breast cancers that express estrogen receptor alpha (ER+) are the most common subtype of breast cancers. Although surgery and anti-estrogen therapies are successful for most of these patients, treatment-resistant ER+ metastatic cancers account for the majority of breast-cancer-related deaths. Immunotherapies have shown promise for other cancer types, but these have been much less effective for ER+ breast cancers. In this review, we update progress in our understanding of the immune microenvironment and the community of other cells that surround ER+ cancer cells at the primary and metastatic sites, the responses of these different cell types to various anti-estrogen therapies, and the net outcomes in experimental and clinical studies. We highlight evolving technologies that will provide greater insight into the biology of ER+ breast cancer and the foundation for new treatment and prevention strategies, in order to reduce mortality of this disease.

**Abstract:**

Estrogen receptor alpha (ERα) marks heterogeneous breast cancers which display a repertoire of somatic genomic mutations and an immune environment that differs from other breast cancer subtypes. These cancers also exhibit distinct biological behaviors; despite an overall better prognosis than HER2+ or triple negative breast cancers, disseminated dormant cells can lead to disease recurrence decades after the initial diagnosis and treatment. Estrogen is the best studied driver of these cancers, and antagonism or reduction of estrogen activity is the cornerstone of therapeutic approaches. In addition to reducing proliferation of ERα+ cancer cells, these treatments also alter signals to multiple other target cells in the environment, including immune cell subpopulations, cancer-associated fibroblasts, and endothelial cells via several distinct estrogen receptors. In this review, we update progress in our understanding of the stromal cells populating the microenvironments of primary and metastatic ER+ tumors, the effects of estrogen on tumor and stromal cells to modulate immune activity and the extracellular matrix, and net outcomes in experimental and clinical studies. We highlight new approaches that will illuminate the unique biology of these cancers, provide the foundation for developing new treatment and prevention strategies, and reduce mortality of this disease.

## 1. Introduction

Breast cancer is one of the deadliest cancers, and is the second leading cause of cancer-related deaths for women worldwide [1]. Cancers that express estrogen receptor alpha (ER+ breast cancers) constitute the most plentiful subset of these cancers, and the importance of estrogenic signals in fueling tumor behavior has led to targeted therapies that are major components of standard treatments. Fortunately, surgery and adjuvant therapies directed at estrogen receptor alpha (ERα) successfully treat the majority of these patients. However, many mechanisms lead to resistance to anti-estrogens [2,3,4,5], particularly in the metastatic setting. Moreover, tumor cells can escape from the primary tumor relatively early in the disease process, likely prior to diagnosis [6,7]. For ER+ disease, metastatic recurrence can manifest decades later, affecting 10–40% of patients, depending on stage at the time of initial diagnosis [8]. Therapy-resistant ER+ breast cancers account for the majority of breast-cancer-related deaths [4,8,9,10], highlighting the need for better treatments.

In established tumors, malignant cells thrive in a complex ecosystem of diverse stromal cells, consisting of heterogeneous fibroblasts, adipocytes, endothelial cells, and immune cells of multiple lymphoid and myeloid lineages in a dynamic extracellular matrix. Although the aberrant neoplastic cells initially elicit an immune response in immunocompetent hosts, surviving tumor cells are edited by this process. In turn, these cancer cells secrete factors that modulate the numbers and activity of immune cells in the local environment, enabling cancer cells to evade the immune system, but also permit immune cells to facilitate invasion and metastasis [11,12,13]. Interfering with communication axes that sustain this immunosuppressed environment to invigorate anti-tumor immunity is the goal of the burgeoning field of immunotherapeutics. For some malignancies, immunotherapies have demonstrated exciting potential for treatment even of advanced disease. For example, immune checkpoint inhibitors (ICIs), such as anti-PD-1, can block inhibitory interactions to reactivate the anti-tumor activity of effector T cells. In patients with immunologically “hot” tumors, characterized by a pre-existing but unresponsive anti-tumor immune response, ICIs can restore anti-tumor activity, resulting in durable tumor regression [14,15,16]. However, application of these approaches to breast cancers, especially ER+ breast cancers, has been disappointing. In general, these tumors have low rates of somatic mutation, express few neo-antigens and contain few infiltrating lymphocytes, indicators of immunologically “cold” cancers [17,18,19,20,21]. A deeper knowledge of the underlying biology and signaling milieu of ER+ cancers that result in the lack of anti-tumor immune activity and general immunosuppression, leading to new ideas, is urgently needed.

Understanding the microenvironments of diverse ER+ tumors in the primary and metastatic settings, and how standard-of-care therapies sculpt the immune microenvironment, is essential to move toward coopting the immune system to control disseminated ER+ disease. Estrogen activity is the best-studied driver of this disease and primary therapeutic target. Estrogens from endogenous sources or supplemental hormone replacement therapy may be elevated prior to diagnosis, and may rise after cessation of long-term adjuvant anti-estrogen therapy. Strategies to reduce or antagonize estrogen activity are the cornerstones of therapies for this disease. Although designed to reduce ERα-mediated signals on the tumor cells themselves, it is now clear that multiple relevant cell types are estrogen-responsive, including immune cell subpopulations, cancer-associated fibroblasts, and endothelial cells, both in the local tumor microenvironment (TME) and elsewhere. Moreover, many of these cells express not only ERα, but also ERβ, and/or the membrane G-protein coupled estrogen receptor (GPER). Although endogenous estrogens are potent agonists at all of these receptors, the clinical anti-estrogens that constitute our chief therapeutic strategies for this disease are diverse (selective estrogen receptor modulators (SERMs), selective estrogen receptor downregulators (SERDs), aromatase inhibitors (AIs)). These agents elicit different spectra of activities at these receptors, which may trigger distinct consequent compensatory mechanisms and divergent net outcomes.

It has long been recognized that estrogen has complex effects in inflammation [22,23,24]. Similarly, although estrogen activity drives proliferation of ER+ breast cancer cells, a major mechanism underlying the efficacy of anti-estrogen therapies, estrogen activity at cancer cell targets can also promote differentiation and inhibit the epithelial mesenchymal transition, increase intercellular adhesion and thereby reduce invasion, and may reduce cancer stem cell activity [25,26,27], all consistent with anti-tumor activity. However, as noted in the references above, many experiments were performed in relatively simple in vitro systems which sometimes yielded different outcomes depending on the model and conditions. In addition, clinically, estrogen and some antiestrogens are bone-protective and this is associated with decreased bone metastases, possibly by estrogen inhibition of osteolysis [28,29]. This complexity has contributed to our incomplete picture of how endogenous and therapeutically manipulated estrogen activity mold the community of different cell subpopulations that comprise the microenvironment of ER+ breast cancers in vivo, with implications for metastatic dissemination, colonization, treatment responses, dormancy, and recurrence.

Here we build on recent reviews of estrogen action in various stromal populations [23,24,30,31,32]. We update progress in our understanding of the stromal cells populating the microenvironments of diverse primary and metastatic ER+ tumors, the distinct estrogen receptors that mediate effects of the range of estrogenic ligands and modifiers of estrogen synthesis to which these cells may be exposed over the course of disease development and treatment, and the effects of estrogen activity on local communication networks to modulate immune activity and properties of the extracellular matrix in the ER+ tumor environment. We summarize recent experimental and clinical studies, and highlight new approaches that will illuminate the unique biology of these cancers, provide the foundation for developing new treatment and prevention strategies, and reduce mortality of this disease.

## 2. Local Immune Environments of Clinical ER+ Breast Cancers

Unlike tumors such as melanomas with copious infiltrated immune cells, breast cancers, like many other solid tumors, are considered immunologically “cold”, with relatively few infiltrating immune cells and little evidence of anti-tumor activity. Most primary breast tumors, especially ER+ cancers, are “immune excluded”, with the majority of immune cell subpopulations in the stroma outside of the tumor cell nests [20,33,34,35].

**Figure 1:**
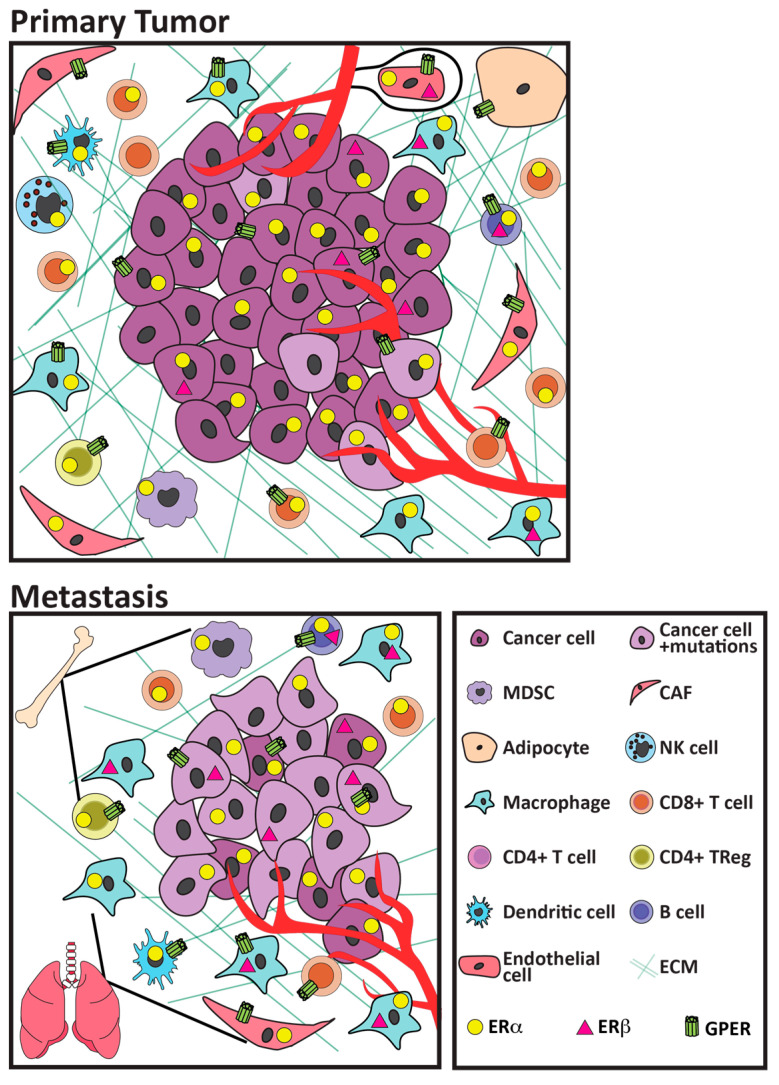
Schematic distribution of estrogen receptors in the microenvironments of primary and metastatic ER+ cancers. Multiple immune cells and other stromal cells in addition to the cancer cells themselves can express multiple distinct estrogen receptors, with different responses to anti-estrogen therapies (see Section 2, Section 3 and Section 4). Top, microenvironment of primary ER+ cancer, enriched in macrophage populations. Bottom, metastatic ER+ lesion, which is enriched in tumor cells selected/evolved for the site, with larger macrophage and reduced CD8+ cell subpopulations. (Data compiled from [24,39,40,41,42,43]). Note that these cell populations exhibit a spectrum of functional states which are likely to display changing levels of receptor expression. Cell responses would be determined by estrogenic ligand, relative numbers of receptors, cell-specific co-activators, and environmental cues.

As described below, even within the context of breast cancers, ER+ cancers exhibit less abundant immune infiltrates, and generally develop fewer somatic mutations with concomitant reduced potential for tumor neo-antigens [36,37,38], compared to HER2+ and triple negative breast cancers (TNBC). Moreover, ER+ disease is very heterogeneous, ranging from luminal A to the more aggressive luminal B and more recently described basal-like tumors. Because luminal A tumors are the most abundant breast cancer subtype, studies which pool all ER+ cancers are skewed in favor of these cancers. Ongoing efforts are beginning to distinguish these subtypes, with implications for treatment options. However, considerably less is known about the milieu of metastatic sites, which is essential for treatment of advanced disease. Importantly, many of these immune cell types have been shown to express some complement of estrogen receptors (shown in Figure 1; functional responses of individual receptors to anti-estrogen therapies shown in Table 1 and discussed in Section 4), and estrogen has been shown to impact aspects of their function (reviewed in [23,24,30,31,32]). However, the majority of functional studies have not been performed in the context of cancers. Nonetheless, as discussed in Section 5, estrogen action on ER+ cancer cells, as well as endothelial, fibroblastic, and immune cell targets, can modulate expression of multiple cytokines and chemokines, which is predicted to alter the numbers and/or activities of immune cell subpopulations.

### 2.1. Immune Microenvironment of Primary ER+ Tumors

T cells and macrophages comprise the vast majority of breast-tumor-associated immune cells, with fewer natural killer (NK) cells, B cells, neutrophils, basophils, and dendritic cells [44,45]. Clinically, however, “tumor infiltrating lymphocytes” (TILs) are often quantified in breast as well as other cancers. These diverse small mononuclear cells are easily recognizable on hematoxylin and eosin-stained sections, and consist of mostly T cells with smaller numbers of B cells. Despite the heterogeneity of the cells detected by this method, the abundance of these predominantly T cell subpopulations correlates with potential anti-tumor immunity. In conjunction with expression of the immune checkpoint proteins PD-L1/PD-1, TIL frequency has been used to identify patients with significant numbers of exhausted T cells who may benefit from immune checkpoint inhibitor therapies for many cancers.

ER+ breast cancers generally exhibit lower TIL infiltration and lower PD-1/PD-L1 expression than TNBC and HER2+ cancers. Further, TIL abundance in ER+ tumors does not correlate strongly with prognosis, in contrast to TNBC and HER2+ cancers, where the abundance of TILs is associated with improved recurrence-free survival and better responses to neoadjuvant treatment and immune checkpoint inhibitors [21,46,47,48,49]. Consistent with the lower lymphocytic infiltrate and PD-1/PD-L1 expression, patients with ER+ tumors respond more poorly to immune checkpoint inhibitors than other breast cancer subtypes (Keynote-028, JAVELIN trials) [50,51]. However, the partial responses observed in a small number of these patients suggest that a more nuanced examination of the spectrum of ER+ tumors might reveal patients who may respond to inhibition of immune checkpoints [50,52].

Although TIL abundance conveys useful clinical information, it masks the heterogeneity of these lymphocytic subpopulations and their functional states. CD8+ cytolytic T cells, an important cell type in TILs, can exhibit a broad range of potential activation/exhaustion/memory states [53]. Recent single-cell analysis of T cell populations across breast cancer cell subtypes adds depth to our knowledge [44,45]. ER+ cancers exhibit lower diversity of the T cell receptor repertoire, consistent with the paucity of neoantigens in these tumors, compared to TNBC and HER2+ cancers [54]. However, luminal B but not luminal A cancers contained large numbers of clonally expanded T cell populations. Interestingly, luminal B tumors displayed distinct T cell-evasive mechanisms from TNBC tumors, with higher frequencies of immunosuppressive immune populations, such as Tregs and M2-polarized macrophages (see below), rather than expression of coinhibitory receptors and ligands, such as checkpoint inhibitors, to suppress T cell-mediated anti-immune responses. These insights provide support for investigation of other immune-driven therapeutic approaches.

Intratumoral T cell subpopulations also include various CD4+ T cell subpopulations, such as immunosuppressive FOXP3+ Tregs [44,55]. These cells reduce anti-tumoral immunity via inhibitory signals to CD8+ T cells, macrophages, and natural killer cells [56]. In contrast to the equivocal predictive utility of total TILs in ER+ cancers, greater numbers of Tregs do correlate with worse outcomes [57,58,59].

Gamma delta T cells (γδ T cells) are a heterogeneous subset of T cells that express γδ T cell receptors, rather than αβ. Unlike αβ T cells, they do not require MHC class I or II peptide presentation, and so can be activated without antigen-presenting cells. Although they can be protective in carcinogenesis, they can be functionally reprogrammed in the tumor environment with net pro-tumor activity, coordinated by secreted Th17 cytokines acting on other immune cell subpopulations, such as MDSCs [60,61,62]. γδ T cells are relatively more frequent in breast cancer than other solid tumors [63], but their presence in different subtypes is less studied. Like natural killer cells, they are recognized as potential immunotherapy tools because they do not require tumor neoantigens to display anti-tumor activity [64].

Although comprising a smaller proportion of TILs, intratumoral B cells are now receiving more attention in the context of breast cancer. These components of the adaptive immune system can confer humoral immunity to tumor antigens, and contribute to antibody-dependent cell-mediated cytotoxicity mediated by natural killer cells. Like T cells, they exhibit a range of activation/differentiation/memory states (reviewed in [65,66]). Similar to other lymphocytic populations, B cells are present at lower numbers in most ER+ tumors compared to HER2+ and TNBC cancers, with the exception of rapidly proliferating luminal B cancers, and any association with prognosis is weaker. However, their frequency is associated with a better prognosis [55]. In contrast, inhibitory Bregs suppress the anti-tumor response, although less is known about this immune population in ER+ breast cancer [67].

Cells of the myeloid lineage, particularly macrophages, comprise the largest immune subpopulations within the ER+ TME, like other breast cancer subtypes [44,45,55,68,69], and many clinical ER+ tumors express high levels of macrophage chemoattractants, such as CCL2 and CCL5 [70]. Extensive studies of tumor-associated macrophages across multiple cancers have shown that these functionally plastic cells can alter the TME by multiple mechanisms, including not only modulation of the inflammatory environment by cytokine production and remodeling of the extracellular matrix, but also supporting angiogenesis, tumor cell invasion, intravasation and metastatic seeding [71,72,73,74,75,76]. Like the other immune cell types in the tumor environment, macrophages can exhibit a broad range of activities. The simplified classifications of M0 (uncommitted), M1 (classically activated inflammatory), and M2 (alternatively activated) polarization states were originally defined based on models of infectious disease (for review, [77,78]). However, it is now clear that this distinction is more nuanced in cancers, including breast cancer, and macrophages exhibit a spectrum of these polarization states [44,72,79]. In general, an M1 signature, characterized by secretion of cytokines such as TNFα, IL-1β, and IL-6, promotes inflammation. In contrast, M2 polarization, characterized by secretion of cytokines such as IL-10 and TGFβ, supports an immunosuppressive environment. Analysis of tumors in the TCGA and METABRIC databases suggested that ER+ cancers were more likely to express an M2 signature than TNBCs, which displayed higher M1 polarization [66], supported by single-cell RNA sequencing analyses [80]. Among the patients with ER+ disease, tumors where macrophages predominantly expressed an M1 phenotype had a better prognosis [81], whereas the M2 marker, CD163, was associated with poorer survival [55,82]. Interestingly, outcomes of crosstalk between TNBC and ER+ tumor cell lines and monocytes in defined in vitro systems were qualitatively distinct [83,84]. In a study examining responses of ER+ primary cancers to neoadjuvant chemotherapy, macrophage content did not vary among luminal A, B and basal subtypes, but an M1 signature was associated with a more favorable response to treatment, and an M2 signature was increased post-chemotherapy, suggesting that targeting this population may provide additional benefit [52].

Although the other immune cell subpopulations described below are present at lower frequencies [44,45], they play important roles in regulating aspects of anti-tumoral immunity/suppression.

Myeloid-derived suppressor cells (MDSCs) are heterogeneous immune populations, which can be derived from either the monocytic (mMDSC) or granulocytic (gMDSC or PMN-MDSC) lineages (reviewed in [85]). Tumor-secreted factors, such as G-CSF, GM-CSF, CCL2, and VEGF, expand ERα-expressing myelocytic precursors in the bone marrow and recruit these immature cell populations to the cancer microenvironment [42,86]. In the local tumor environment, their suppressive activity can be stoked by cytokine signals, and they potently inhibit anti-tumor immunity, including not only T cell cytotoxic activity, but also B and natural killer cell function. As for many other immune cell subpopulations, MDSCs are generally lower in ER+ breast cancers than other breast cancer subtypes, but higher in luminal B compared to luminal A tumors [44,87,88]. Higher frequencies correlate with poorer overall survival for ER+ cancers [89], and circulating mMDSCs tended to correlate with worse overall survival in patients with metastatic ER+ disease, although the association was more robust in ER-negative breast cancers [90]. In contrast to the accumulating evidence for the importance of immature intratumoral neutrophils in ER+ breast cancer, less is known about mature neutrophils in these tumors, although they are higher in TNBC [44,91].

Dendritic cells are another myeloid subset which plays important roles in activation of T cells by expression of co-stimulatory molecules for antigen presentation and secretion of immunomodulatory cytokines. These cells are relatively rare in the TME, but like other immune subpopulations, they are sculpted by local cues. Studies of the transcriptomes of dendritic cell subsets from various breast cancer subtypes demonstrated that these cells from ER+ cancers exhibited lower activation of the Type 1 interferon pathway than those from TNBCs. However, the activation signatures for different dendritic cell subsets correlated with prognosis in ER+ disease [92].

Natural killer (NK) cells are components of the innate immune system which can kill tumor cells independent of antigen recognition, in contrast to the adaptive immunity provided by T cells. They can induce apoptosis by exocytosis of perforin and serine proteases, or by antibody-dependent cell-mediated cytotoxicity, and also secrete cytokines to modulate the immune environment [93,94]. In ER+ tumors, NK cell frequency declines with tumor progression; greater numbers are associated with a better prognosis [55]. Because they can display potent anti-tumor activity in the absence of tumor neoantigens, they are recognized as potential immunotherapy tools [94].

Much of our current knowledge is based on immunocytochemistry, flow cytometry, and molecular analyses of heterogeneous tumor homogenates of smaller numbers of patient tumors, supported by deconvolution and interrogation of transcriptomes in publicly available data sets. However, single-cell RNA sequencing and mass cytometry of patient tumors are beginning to refine our understanding of the immune landscape [34,44,45,68]. These studies confirm the paucity of CD8+ T cells and abundance of myeloid cells in ER+ cancers, with fewer NK cells, B cells, granulocytes, plasma cells, basophils, and plasmacytoid dendritic cells, and reveal the continuum of differentiation states, particularly of intratumoral T cells and myeloid-derived immune cell subpopulations.

Together, these studies indicate that ER+ breast cancers generally contain fewer immune cells, particularly CD8+ cytotoxic T cells, and suggest that the frequency of immune subpopulations with high prognostic value for other cancer types, including TNBC, are less valuable for ER+ cancers. Studies are beginning to delineate differences among ER+ cancers. These reports also suggest that the dominant immunosuppressive milieu might be mediated by mechanisms other than expression of immune checkpoint programs, and point toward other cell subpopulations, such as macrophages and Tregs, as possible therapeutic targets. Notably, many of these immune cells express estrogen receptors (Figure 1), indicating that they, too, can directly respond to endogenous estrogens, and therapeutic estrogens and anti-estrogens. Moreover, estrogen activity has been shown to modulate chemokine/cytokine networks that influence anti-tumor immune activity using in vitro models, as discussed in Section 5.

### 2.2. Influence of Somatic Mutations in Oncogenic Drivers and Anti-Estrogen Resistance

ER+ breast cancers exhibit considerable variation in immune cell content, which is not surprising given the range of somatic mutations, gene expression patterns, and biological behaviors within this breast cancer subtype [36,37]. Tumor intrinsic features including genotype are known to influence the tumor immune infiltrate [18,95]. As discussed in Section 2.1, reports are only beginning to parse out any differences in the tumor immune microenvironment among different subtypes of ER+ tumors.

Many of the signaling pathways commonly dysregulated, particularly in clinical anti-estrogen-resistant ER+ breast cancers, are known to potently modulate the surrounding immune environment. Increased activity of the phosphatidylinositol 3-kinase pathway was associated with increased FOXP3+ cells, and mutations in *PIK3CA* were associated with more abundant CD8+ lymphocytes, associated with a higher risk of recurrence [96]. The RAS pathway is strongly activated in many aggressive ER+ luminal B breast cancers, either due to mutations in *KRAS*, or more commonly, downregulation of Ras-GAP proteins [97,98,99,100,101]. This activation alters recruitment and differentiation of immune cells, including macrophages, MDSCs, and Tregs, to promote an immunosuppressive TME, via NFκB-dependent production of TNFα, IL-1α,β, IL-6, CCL2, ligands of CXCR2, and NFκB-independent increases in TGFβ, IL-10, and GM-CSF [102,103,104].

Interestingly, Ellis and colleagues recently reported that tumors which exhibited de novo resistance to neoadjuvant aromatase inhibitors (defined as luminal B) contained higher transcripts for induction of immune tolerance and inhibition of T cell activation, including increases in mRNAs encoding the targetable checkpoint inhibitors, IDO1, PD1, and LAG3 [69]. These markers were associated with activation of the IFNγ-STAT1 pathway. IDO1 was highly expressed on intratumoral myeloid cells, and this expression was associated with PD-L1 expression on carcinoma cells, and PD-1, LAG3, and FOXP3 expression in TILs. Notably, this immune pattern was associated with larger macrophage populations, and the association was highest with M1, rather than M2 or M0 signatures.

Together, these findings underscore the heterogeneity of immune populations across diverse ER+ primary cancers and the link between immune activity and tumor behavior, and begin to establish markers to identify patients who may benefit from immune-targeted therapies.

### 2.3. Differences between the Microenvironments of Primary and Metastatic ER+ Tumors

Understanding the environment of disseminated disease is critical for successful treatment of advanced ER+ disease. The metastatic environment and differences from that of the primary tumor are understandably harder to study. Not only are metastatic sites generally more difficult to biopsy, but frequently much time has elapsed since the surgical removal of the primary tumor and the appearance/treatment of disseminated disease. Further, many patients in early-phase trials who have contributed to these data have been treated with numerous agents, including with multiple anti-estrogens, since the initial diagnosis. Despite these obstacles, it has been shown that many ER+ metastases acquire additional somatic mutations, including those which would impact the immune TME [99,100] (see Section 2.2). Deconvolution of transcriptomes from matched metastases and primary tumors across breast cancer subtypes revealed that distant sites displayed fewer CD8+, Treg, and dendritic cells, but increased numbers of macrophages, particularly those exhibiting M2 polarization, although ER+ cancers showed smaller differences [105,106,107]. Emerging studies are only beginning to evaluate differences among metastatic sites. Bone lesions, which comprise 60–70% of recurrent ER+ disease [108,109,110], are more difficult to obtain and are generally not well-represented in many studies. However, a recent report deconvoluting transcriptomic data showed that immune cell composition varied with metastatic site, with bone displaying greater numbers of neutrophils [111].

ER+ metastases that have acquired mutations in the ligand-binding domain of ERα (mESR1) leading to constitutive activation, most commonly in response to treatment with aromatase inhibitors, exhibited differences in the immune cell complement from metastases expressing wildtype ERα, including increased Tregs and PD-L1-expressing macrophages, and increased activity of the Type 1 IFN pathway [107]. The extent of these changes suggested that acquisition of these mutations is associated with multiple compensatory pathways, apart from direct gene targets of ERα.

Extending our knowledge of immune cell subpopulations, their functional states, and the roles that these cells play in dormancy, recurrence, and treatment susceptibility of ER+ metastatic disease in different locations, is a critical area of need. A recent study suggested the intriguing possibility that a T cell subset may mediate tumor dormancy [112].

### 2.4. Bone as a Metastatic Site

Between 60 and 70% of metastatic recurrences of ER+ breast cancer are in bone; a much larger proportion than for other subtypes of breast cancer [108,109,110]. Multiple resident cell types (osteoclasts, osteoblasts, as well as other stromal cells, including immune cells) are estrogen-responsive [28,113], providing a unique site for ER+ metastatic lesions. The mechanisms that underlie the seeding of disseminated ER+ tumor cells at this site, the prolonged dormancy frequently observed, the development of overt metastatic lesions from dormant cells, and the role of estrogen in these processes are not well-understood (reviewed in [10,28,113,114]). Estrogen opposes overt metastatic outgrowth, mediated by ERα and perhaps also ERβ in osteoclasts and osteoblasts, by mechanisms that also protect bone from osteoporosis prior to menopause. The RANK/RANKL signaling pathway triggers osteoclast differentiation and consequent osteolysis, which increases cytokines that may promote tumor growth. Estrogen opposes this pathway by increasing expression of osteoprogeterin, a soluble decoy receptor for RANKL, and by suppressing osteoclast differentiation. Some SERMs, including tamoxifen, are agonists in bone, and also reduce bone resorption (Section 4.1, [115,116]). However, some chemokines which are induced by estrogen in ER+ breast cancer cells (Section 5.1) may not be beneficial. For example, CCL2 increases RANK expression in osteoclast progenitor cells [117]; CXCL12 has been implicated in homing of ER+ tumor cells to bone [118]. These observations suggest that estrogen can amplify regulatory axes which could promote bone metastases. Moreover, the bone environment can epigenetically alter ER+ tumor cells to promote resistance to anti-estrogens [119]. The distinct actions of estrogen and interplay among the multiple estrogen-responsive cell types predict a complex response to anti-estrogen therapies. In light of the importance of the bone environment in devastating recurrences of ER+ disease, a better understanding of estrogen actions in this niche remains a critical need.

### 2.5. Summary

ER+ breast cancers exhibit fewer somatic mutations and lower numbers of infiltrated lymphocytes than other breast cancer subtypes, resulting in a highly immunosuppressed environment in the majority of these tumors. Clinical studies have demonstrated that releasing inhibition of exhausted T cells with immune checkpoint inhibitors has little benefit for most of these patients, underscoring the need to directly address the suppressive environment conferred by macrophages, mMDSCs, gMDSCs, and Tregs. The tumor cells themselves are diverse. Only 1% of tumor cells need to express detectable ER+ to be considered ER+ breast cancers [120], and recent single-cell analyses have illuminated the heterogeneity of the tumor parenchyma and local stromal environments [44,45,68,80]. Although total TILs were not informative in ER+ breast cancers, spatial distribution was predictive of response to anti-estrogen therapies and prognosis, highlighting the importance of heterogeneity of the tumor cells and local microenvironments, including cytokine networks and associated immune cell populations [121]. Additional application of single-cell sequencing, multiplex fluorescence, and developing technologies which enable spatial resolution of cell identities, functional states, and gene expression in cellular niches within the TME of diverse clinical ER+ breast cancers at the primary and different metastatic sites, especially bone, will illuminate the underlying biology.

Notably, many of these immune populations have been shown to be estrogen-responsive (reviewed in [23,24,30,31,32], Figure 1), albeit in experimental systems other than cancers. Endogenous estrogens and therapeutic anti-estrogens would be predicted to directly modulate the activity of these cells, as well as indirectly influence their activity via cytokines/chemokines secreted from other nearby cell types (Section 5), and assessment of the net effects of these therapies in experimental and clinical cancers supports this (Section 6). As discussed in Section 3, features of the extracellular matrix are closely related to inflammation, and estrogen participates in the regulation of these features, as well.

## 3. Deposition, Composition, and Architecture of the Extracellular Matrix

Tumorigenesis is associated with marked changes in the composition and structure of the surrounding extracellular matrix, and the resulting increased density and stiffness can alter tumor stem cell activity, migration, metastasis, and immune activity (reviewed in [76,122,123,124,125]). Matrix proteins and remodeling enzymes are synthesized by many cell types in the TME, including not only heterogenous fibroblast populations, but also tumor cells and some immune cells, such as macrophages and even T cells [76,126,127,128]. Mechanical interactions with tumor cells and altered protein composition, including increases in collagens and matrisomal proteins, such as TNC, FN1, and VCAN, as well as cross-linking and matrix-degrading enzymes, such as LOX and matrix metalloproteinases (MMPs), functionally reorganize the matrix in the local environment [124,129,130]. Moreover, components of the extracellular matrix can precondition the metastatic niche [125,131,132,133]. Signaling pathways that are commonly activated in aggressive ER+ breast cancers, such as RAS, have been linked to increased fibrosis in other cancers [103,134].

Conversely, these alterations in the surrounding extracellular matrix can exert dramatic effects on multiple cellular components of the TME. In addition to altered signaling pathways in the tumor cells themselves, such as increased signaling through FAK and downstream pathways [122,135,136,137], the biomechanical and chemical signals modulate the inflammatory environment, including macrophage and T cell activation [76,127]. Matrikines, bioactive protein fragments produced during matrix remodeling, may act as chemokines or cytokines [138]. Mechanical signaling through YAP-TAZ in tumor cells can not only drive aggressive behavior [139,140], but can also reduce anti-tumor immune responses by increasing expression of the checkpoint ligand, PD-L1, and chemokines that recruit MDSCs [141,142]. In immune cells, this pathway can inhibit anti-tumor immunity by promoting differentiation of Tregs and M2 polarization of macrophages [141,142]. The physical matrix structure also plays a role in the spatial distribution and trafficking of cells in the TME (reviewed in [125,138]). For example, immune cells can migrate along Type 1 collagen fibrils; a dense matrix restricts infiltration of T cells into the tumor nests. The latter can impede drug delivery, a particular consideration for immunotherapies [143].

Although HER2+ and TNBC subtypes are considered to be more fibrotic [128], the organization of fibrillar collagens in the microenvironments of ER+ cancers is linked to biological behavior and is an independent prognostic indicator of disease-free survival [144]. Primary ER+ tumors that exhibit regions where the surrounding collagen fibers are aligned perpendicular to the tumor surface (Tumor Associated Collagen Signature-3; TACS-3) [145]) predict significantly worse patient outcomes. Consistently, aligned collagen in patient tumors correlates with elevated pro-inflammatory COX-2 and CD163+ (M2) macrophages, and poor prognosis [146]. Moreover, high expression of MMP-9 in CD163^+^ tumor-associated macrophages was associated with worse overall survival in ER^+^ tumors (*p* < 0.001) but not in ER-negative cancers [82]. The link between fibroblast characteristics and anti-estrogen responsiveness of ER+ clinical cancers further supports the interaction of these cell types in the ER+ TME [147].

Like immune cell components, the increasingly acknowledged importance of the extracellular matrix has motivated the development of therapies that target components of the matrix, CAFs [76,125,148,149,150], and the YAP-TAZ pathway [142]. The heterogeneity of fibroblasts near the primary tumor [126,151] and in the axillary lymph node [152] supports the complex roles of these cells in disease progression. As discussed in Section 5, estrogen action on multiple cell types can modulate synthesis of components which would alter properties of the extracellular matrix. Moreover, estrogen and anti-estrogen therapies can impact both the composition and architecture of the extracellular matrix in experimental models in vivo, likely by acting on both tumor epithelia, macrophages, and CAFs via multiple receptors [153,154,155] (Section 6.1). Indeed, as discussed in Section 6.2, transcriptomic analyses of clinical tumors treated with aromatase inhibitors in a preadjuvant setting showed that the HIPPO pathway was among the most affected. These effects together with the close links to inflammation underscore the importance of these non-tumor cellular components of the ER+ breast cancer microenvironment.

## 4. Estrogen Receptors

Whether pre- or post-menopausal, patients with ER+ breast cancers may be exposed to endogenous estrogens from multiple sources prior to diagnosis. In pre-menopausal women, the ovaries and if pregnant, the placenta, synthesize copious estrogens to coordinate processes critical for fertilization and pregnancy. In post-menopausal women, adrenal androgens aromatized in breast adipose cells can raise local estrogen exposure in the breast environment, and any exogenous hormone replacement therapies would raise systemic estrogen activity.

Reduction of estrogen activity is the backbone of the therapeutic and prevention strategies for ER+ breast cancer. The SERM, tamoxifen, is a standard adjuvant therapy in premenopausal women [8,115], aromatase inhibitors are frequently used to prevent recurrence of ER+ disease in post-menopausal women [156,157], and the SERD, fulvestrant, is a prominent second-line treatment in advanced disease [158]. After cessation of these therapies, endogenous estrogen activity can rebound. Thus, patients, particularly those with metastatic disease, are likely to be exposed to varying combinations of endogenous estrogen and anti-estrogen activities over the course of tumorigenesis and treatment.

Three distinct receptors (as well as splice variants, and palmitoylated receptors localized to the plasma membrane) can mediate the actions of estrogenic ligands in diverse target cells to modulate ER+ breast disease. As shown in Figure 1, these receptors have been reported to be expressed in the multiple cell types that comprise the microenvironments of ER+ cancers. Target cell responses are dictated not only by the repertoire of expressed receptors, but also target cell specific coactivator abundance and gene promoter availability, which may evolve as cells respond to environmental cues. As summarized below and shown in Table 1, endogenous estrogens and therapeutic anti-estrogens have distinct spectra of effects on these different receptors.

### 4.1. ERα

Expression of ERα distinguishes ER+ breast cancer from other breast cancer subtypes. Clinical tumors exhibit considerable range in the proportion of cells that express detectable ERα protein; the current cutoff in clinical laboratories is 1% [120]. The best-studied outcome of estrogen action, proliferation of tumor cells, has been the endpoint for development of clinical anti-estrogens. Tamoxifen, first in the class of SERMs and still a major component of treatment for premenopausal disease, usually behaves as a competitive antagonist in breast cancer. However, like other SERMs, its complex context-dependent actions can lead to agonistic actions [3,115,154]. Other SERMS, such as raloxifene, are also competitive inhibitors, and display a different spectrum of agonist activities across different normal cell types [116]. Although SERDs, such as fulvestrant, also competitively inhibit endogenous estrogenic ligands, they trigger rapid degradation of ERα and do not display agonist activity. While fulvestrant has been the primary compound in this class, orally available compounds with similar activity and improved bioavailability are being developed (e.g., [159,160]). In contrast, AIs, such as letrozole and anastrazole (nonsteroidal AI), or exemestane (steroidal AI), do not bind to ERα, but rather reduce endogenous estrogens by inhibiting aromatase, which converts androgens to estrogens.

However, this is more complex than it first appears. Even the responses of well-characterized ERα breast cancer cell lines to 17β-estradiol in vitro are context-dependent. The estrogen-stimulated ERα cistrome is dramatically altered by growth factors [161], progesterone [162], androgens [163], and important for modulation of the immune environment, the cytokine milieu [164,165]. Notably, inflammation, such as initiated by elevated TNFα, can modulate the target transcriptome [166,167,168]. TNFα and IL-1β can initiate signals leading to phosphorylation of ERα at p305, resulting in ligand-independent activation [169]. Thus, responses in vivo would be highly dependent on the local environment.

Different ligands may interact differently with these modifying signals. A recent report suggests that estrone, the prevalent endogenous estrogen after menopause, may more potently stimulate NF-κB-mediated cytokine expression [170], key players in inflammation and cancer [171]. Exposure to tamoxifen may increase NF-κB signals [172]. Further, somatic mutations common in aggressive ER+ tumors, such as those leading to increased signals through Ras pathways, also increase NF-κB signals [173].

In addition, prolonged AI treatment can result in point mutations in the ligand binding domain of *ESR1* (mutant ESR1, mESR1), particularly in metastatic tumors, which result in constitutive activation (reviewed in [4,164,174]). These mutations are associated with alterations in transcriptional profiles beyond ER target genes, as well as different immune composition of the tumors [175,176]. Although AIs are no longer effective, SERDs, such as fulvestrant, can degrade these constitutively active receptors [5], and other SERMs are under development, e.g., [177]).

Finally, alternatively spliced ERα variants exhibit dominant negative activity (e.g., [178]). Tumors with high expression of these variants display transcriptomes more similar to basal tumors, and they respond to therapies as ER- tumors [179].

### 4.2. ERβ

ERβ is highly homologous to ERα, and regulates overlapping but distinct target genes [115,180]. About 30% of ER+ breast cancers also express ERβ [181,182]. ERβ can homodimerize, or heterodimerize with ERα, and thus relative levels are important [183]. Transfecting ERβ into MCF7 cells, which express endogenous ERα, enhanced or counteracted ERα actions on distinct subsets of target genes. Activation of ERβ generally suppresses estrogen-induced proliferation in the context of ERα [184,185].

Not surprisingly, the differences in the ligand-binding domains of ERα and ERβ confer different responses to endogenous and therapeutic anti-estrogens, which also can differ with cell type, complement of co-regulators and promoter context [115,186]. For example, tamoxifen can activate ERβ [187], and fulvestrant does not result in degradation of ERβ when co-expressed with ERα [188].

The role of ERβ and its splice variants in ER+ breast cancer remains poorly understood. In the breast cancer niche, ERβ is also expressed in cells other than tumor epithelium, including stroma and endothelium [24,181], and in the bone metastatic niche, both osteoclasts and osteoblasts [28]. The significance of this expression is relatively underexplored.

### 4.3. G Protein Coupled Estrogen Receptor (GPER)

GPER is a membrane receptor which mediates rapid estrogen-induced non-genomic signaling via transactivation of EGFR (reviewed in [43]). In addition to endogenous estrogens, clinical antagonists (tamoxifen, fulvestrant) also activate GPER, potentially complicating responses to SERM and SERD endocrine therapies (Table 1).

Although GPER can be expressed on multiple cell types which impact ER+ disease, including ER+ tumor cells themselves, CAFs, endothelial cells, and immune subpopulations, its role in breast cancer is not well-understood [189,190,191]. In CAFs, GPER-mediated signals can upregulate IL-1-driven inflammatory pathways and angiogenic factors [43,192]. However, tamoxifen activation of these receptors can inhibit differentiation of these cells in non-breast cancers, reducing deposition and remodeling of the surrounding extracellular matrix [193]. GPER activation can also influence T cell populations, including increasing immunosuppressive Tregs [194], and decreasing inflammatory IFNγ production [43]. A recent intriguing study demonstrated anti-inflammatory activity in the trophoblast cells during pregnancy; activation of GPER by placental estrogens reduced Type 1 IFN signals, thus playing a role in protecting the fetus from maternal immune responses [195]. Investigation of GPER in ER+ breast cancer with increasingly specific ligands will clarify its actions.

### 4.4. Summary

The widespread distribution of distinct estrogen responsive receptors on the cells comprising the environments of ERα+ cancers, shown in Figure 1, illustrates the complexity of potential estrogenic signals within the TME. With the exception of mESR1, which is constitutively active, all of these receptors are robustly activated by 17β-estradiol, but even these responses are strongly modulated by the growth factor/inflammatory milieu. Moreover, other endogenous estrogens, such as estrone, and therapeutic anti-estrogens exert different spectra of activity (Table 1). SERMs, such as tamoxifen, display context-dependent antagonistic or agonist activity, and can activate GPER. SERDs, such as fulvestrant, block all ERα-mediated signals, including mESR1, and like SERMs, they are agonists at GPER. In contrast, AI treatment reveals the absence of ligand-dependent estrogen signaling, including signals to GPER, but does not inhibit growth-factor-activated ligand-independent signals or constitutive signaling through mESR1. Furthermore, AIs also augment signaling via AR, secondary to the concomitant increase in androgens [196]. The outcome of estrogenic action reflects the availability of specific agonists/antagonists, the integration of signals triggered by different receptors expressed on a given cell, the dynamic responsiveness of these cells to other changes in environment, and interplay with different cell compartments.

## 5. Cell Specific Estrogenic Regulation of Genes Which Would Modulate the Immune Microenvironment of ER+ Breast Cancers

A burgeoning literature supports the ability of estrogens to signal to multiple cell types in the microenvironments of ER+ breast cancers, which would include not only tumor cells, but also CAFs and immune populations, supported by the widespread distribution of the various estrogen receptors (Figure 1). Moreover, systemic neoadjuvant or adjuvant anti-estrogen treatment can also affect other sites—e.g., bone marrow, immune cell precursors, fibrocytes, as well as sites of immune cell maturation (e.g., spleen). The relevance of estrogen/antiestrogen actions on non-cancer cells has been demonstrated in experimental models of non-estrogen receptor-expressing cancers, including TNBC, ovarian, and pancreatic cancers [42,155,160]. The effects of manipulation of estrogenic activity on the behavior of stromal cell types relevant to the TME in defined in vitro systems have been comprehensively reviewed [23,30,31,32]. In ER+ breast cancer, these target cells would also include the cancer cells themselves.

Estrogenic ligands can tilt the balance of pro- and anti-tumorigenic inflammatory activity in the TME by altering expression of chemokine ligands and their receptors, other cytokines and growth factors and their receptors, and proteins that remodel the extracellular matrix. However, the scope of these changes, and which of the multiple potential target cells are the source of these factors, are not well-understood. Few studies have interrogated isolated primary cells from breast cancers. For insight into the mediators of estrogen activity, we collected data on estrogen-regulated genes in isolated human or murine cells or cell lines representing cell types that populate the microenvironments of ER+ cancers from published studies and microarray datasets deposited to the Gene Expression Omnibus [197] (Supplementary Table S1). We examined genes encoding CC- and CXC- chemokines and other cytokines, and ECM components and modifiers, proteins that would modulate immune activity and extracellular matrix components of the TME. Although not comprehensive, these results demonstrate the potential for estrogen and anti-estrogens to strongly influence the TME by acting on multiple cell types.

### 5.1. Tumor Epithelia

**Figure 2:**
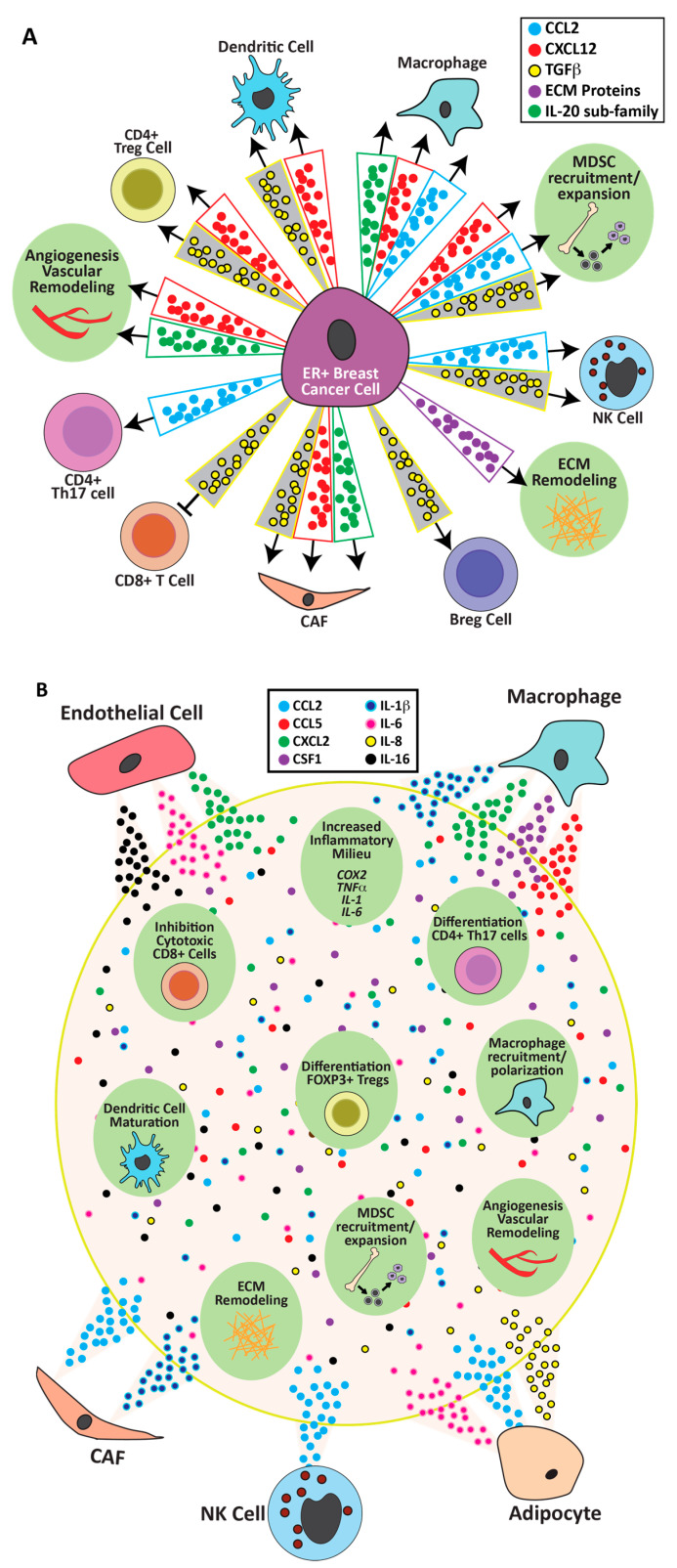
Changes in estrogen potentially can tilt the balance of immune activity by acting directly on multiple cell populations in the ER+ breast cancer environment to modulate the cytokine/chemokine milieu and extracellular matrix (predicted by experimental studies discussed in Section 5). (**A**) Estrogens and anti-estrogens acting on ER+ breast cancer cells impact major communication arcs in the TME. Arrows indicate increased numbers and/or function; blocks indicate inhibition. Agonists increase expression of the molecules shown in the legend, represented by white triangles. Antagonists increase TGFβ, as shown in the shaded triangles. (**B**) Estrogen increases chemokine/cytokine secretion from stromal cell populations (outer ring), which would alter multiple immune processes in the ER+ TME (center). Importantly, these predictions are based on studies in defined model systems, which need significant additional study in the context of diverse primary and metastatic ER+ breast cancers. Moreover, as discussed in the text, estrogen signals can be modified by the cytokine milieu, and the ability of target cells to respond varies with differentiation state and other local environmental cues, which evolve with tumor progression.

The actions of estrogenic ligands and consequences of aromatase inhibitors on the tumor epithelia themselves set ER+ tumors apart from other breast cancer subtypes. The majority of studies of estrogen responses in ER+ breast cancer cells have utilized the MCF7 and T47D cell lines, which were derived from patient tumors and model luminal A cancers. In addition to published papers, seven GEO datasets containing microarray gene expression from MCF7 cells and one from T47D cells were examined for estrogenic transcriptional regulation of genes likely to modulate the ER+ breast cancer TME (Supplementary Table S1). Experimental conditions across these data sets varied and, not surprisingly, expression patterns varied somewhat as well. However, these studies revealed potent estrogenic regulation of several major communication axes that would regulate the inflammatory tumor environment of ER+ breast cancers (Figure 2A).

CXCL12: The ability of 17β-estradiol to robustly increase expression of the chemokine CXCL12 in breast cancer cells was reported by the Korach lab and others [198,199,200], and was confirmed in multiple GEO datasets [201,202,203,204,205,206,207,208] (Supplementary Table S1). Its primary receptor, CXCR4, is highly expressed on multiple stromal cells including CAFs, macrophages, monocytes, dendritic cells, T and B cells, and endothelial cells [86,126,209]. As illustrated in Figure 2A, the central role of the CXCL12-CXCR4 axis positions it to facilitate effects of estrogen activity on cancer progression and metastasis by effects on immune cell recruitment, the vasculature, and CAFs [86,126,209,210,211]. Moreover, expression of CXCL12 and CXCR4 have been reported to have prognostic value for worse overall survival in breast cancer [212,213].

CCL2: CCL2 is highly expressed in clinical ER+ breast cancers compared to normal breast epithelium [70,214]. It promotes recruitment of monocytes, MDSCs, CD4+ Th17 T cells, and NK cells, and is associated with tumor progression [86]. 17β-estradiol increases its transcripts in MCF7 cells [201,208] (Supplementary Table S1), mouse models, and breast tissue explants [70]. In some signaling contexts, these effects are intensified. Estrone more effectively raised levels of mRNAs for CCL2 and IL-6 in MCF7 cells pretreated with TNFα than 17β-estradiol, which was validated in MCF7 tumor-bearing mice treated with estrone [170]. In total, these observations indicate that estrogenic agonists and anti-estrogens can directly regulate expression of this important recruiter of immune cells to the ER+ breast cancer environment.

TGFβ: TGFβ is a major player in the immunosuppressive environment of established cancers [86,215,216,217], in sharp contrast to its potent tumor-suppressive activity in the normal mammary gland [218]. In invasive cancers, this growth factor acts on multiple target cells in the TME to drive tumor progression (Figure 2A). TGFβ interactions with estrogen over the course of mammary development and breast cancer reflect this complex biology, and these factors interact at multiple points in their signaling pathways [218]. TGFβ activity is tightly regulated at multiple levels and, therefore, mRNA levels do not necessarily reflect activity. Interestingly, several studies found that 17β-estradiol reduced mRNAs for TGFβ isoforms in MCF7 cells [199,203,204,206] (Supplementary Table S1). Knabbe and colleagues showed that longer treatment of MCF7 cells with tamoxifen also increased TGFβ, and this signal mediated increased expression of FOXP3, a marker for Treg cells, in anti-estrogen-treated MCF7 cells co-cultured with CD4+ cells [219]. This regulation is reflected in response to supplemental 17β-estradiol in experimental syngeneic models in vivo (Section 6.1) and clinical responses to aromatase inhibitors (Section 6.2).

IL-20 subfamily of cytokines: Interleukins and their receptors, critical molecules in inflammation, were also regulated by estrogen activity in ER+ breast cancer cells. Multiple datasets showed that 17β-estradiol strongly increased transcripts for several members of the IL-20 subfamily of interleukins, including IL-19, IL-20, IL-24, IL-28A, and IL-28B in MCF7 cells [201,206,208] (Supplementary Table S1). Although this subfamily is not well-studied in breast cancer, they are recognized inflammatory mediators in psoriasis and rheumatoid arthritis [220,221]. IL-20 induced M2 macrophage differentiation from bone marrow-derived macrophages in vitro, and IL-20 blockade reduced CD206+ (marker for M2) macrophages in a pancreatic cancer model in vivo [222]. Direct examination will elucidate a potential role for these interleukins in estrogenic modulation of macrophage, CAF, and endothelial function in the environment of ER+ breast cancer TME.

### 5.2. Myofibroblasts/Cancer-Associated Fibroblasts (CAFs)/Immune Cells

Direct effects of estrogen activity in isolated cells are summarized below, and illustrated in Figure 2B.

CAFs: As shown in Figure 1, virtually all cell types that comprise the ER+ TME have been reported to express not only ERα, but also ERβ and/or GPER. Although for many cell types the functional roles of individual receptors and consequences of co-expression have only begun to be explored, GPER function in CAFs from various sources has received more attention (see also [30]). CAFs can express both ERα [223] and GPER [193]; the latter can be activated by clinical estrogen antagonists, including fulvestrant and tamoxifen, as well as 17β-estradiol. 17β-Estradiol signaling through GPER robustly increased expression of IL-1β in CAFs isolated from human breast cancer tumors [192]. Tamoxifen through GPER inhibited differentiation of pancreatic stellate cells into CAFs [193]. Although the mediating receptor was not identified, fulvestrant and tamoxifen decreased secretion of CCL2, but increased IL-6 and IL-10 from the murine mammary stroma of nude mice bearing human MCF7 tumors [224].

Adipocytes: Adipocytes are potent sources of cytokines that can modulate breast tumorigenesis [170,225]. Interestingly, estrone, but not 17β-estradiol, was able to sharply increase mRNAs for CCL2, IL-6, and IL-8 in isolated primary adipocytes [170].

Monocytes/Macrophages: In isolated human monocytes cultured in vitro, 17β-estradiol stimulated and fulvestrant blocked IL-1β, IL-6, CCL2, CCL3, CCL4, and TNFα secretion [224]. In the mouse RAW264.7 macrophage cell line, 17β-estradiol increased transcripts for many chemokines/cytokines, including CCL4, CCL5, CCL12, CCL25, CX3CL1, CXCL2, CXCL10, and CXCL16, IL-1β, TNFα, and CSF-1, as well as those for CCR2, which would augment its response to estrogen-stimulated CCL2 from tumor cells [226] (Supplementary Table S1).

Myeloid progenitors: 17β-estradiol has been shown to act directly on these cells via ERα, resulting in expansion and differentiation of MDSCs and dendritic cells [42,227], which would impact multiple aspects of the relationship between tumor cells and immune activity. Further, CD44+ progenitor cells isolated from bone marrow and treated with 17β-estradiol increased mRNAs encoding CXCL10, IL-16, IL-1α, and IL-1β, as well as CCRL2 [228].

Other immune cell populations: 17β-Estradiol increased CCL2 secretion from human NK cells isolated from first trimester decidua, which was pro-angiogenic in a human endometrial endothelial cell network formation assay [229]. CD4+ cells isolated from mouse spleen and treated in vitro with 17β-estradiol upregulated expression of FOXP3 and IL-10 [230], consistent with strong induction of an immunosuppressive Treg population.

This diverse collection of data from a variety of experimental models illustrates the potential for estrogenic and anti-estrogenic ligands to alter the expression of genes in cells of the TME that will modulate immune responses to the tumor (Figure 2B). In the in vivo ER+ cancer microenvironment, these stromal cells, like the cancer cells, present evolving target cell contexts for estrogen action. Outcomes of estrogenic signals to primary cells isolated from diverse ER+ tumors under controlled hormone conditions await discovery.

### 5.3. Endothelial Cells

Estrogens are established regulators of vascular tone and activity through rapid, as well as genomic, signaling pathways, and published studies have focused on vasoactive compounds, such as eNOS [231,232]. Although vascularization of tumors, including breast cancer, has been extensively studied, effects of estrogen on endothelium from ER+ breast cancers are lacking. Nonetheless, two groups have reported microarray expression data of 17β-estradiol-treated human umbilical vein endothelial cells (HUVEC). Both groups reported widespread responses in genes encoding immune regulators, such as increased CXCL2, IL-6, and IL-16, and reduced IL-20 and IL-21, indicating a potential to shift the local cytokine environment, as well as proteins of the extracellular matrix (discussed below) [233,234] (Supplementary Table S1, Figure 2B). With the caveat that endothelial cell function is specific to the tissue environment [235], these studies illustrate the potential of endothelial cells to alter chemokine activity in response to estrogen receptor agonists.

### 5.4. Multiple Cell Type Contributions to Extracellular Matrix Remodeling

The extracellular matrix is dynamically remodeled in normal tissue function as well as tumor growth, and changes in the composition and architecture of the extracellular matrix facilitate metastasis (reviewed in [76,122,123,124]). As discussed in Section 3, straight collagen fibers which orient perpendicularly to the ER+ tumor boundary (TACS-3) are prognostic for poor outcome [129,145]. Analysis of ECM composition in patient biopsies of invasive ductal carcinomas identified a set of 19 matrisomal proteins that positively correlated with straight collagen alignment [129]. Estrogen activity increased transcripts for many genes related to remodeling of the extracellular matrix in multiple target cell models, including breast cancer cell lines, the RAW264.7 macrophage model and the endothelial HUVEC. Upregulated transcripts included collagens, matricellular proteins, and various endopeptidases, including cathepsins and metallopeptidases (MMPs) (Supplementary Table S1). Although fibrillar collagens were not strongly affected, 17β-estradiol robustly increased mRNAs for THBS1 and COL12A1, components of the signature of aligned collagen in clinical samples [129], in multiple ER+ breast cancer cell line datasets [201,204,205,206]. Moreover, mRNAs encoding COL12A1 and several MMPs were increased by estrogen in the RAW264.7 macrophage cell line and HUVEC model [226,233] (Supplementary Table S1). Together, these data indicate that estrogen agonists can drive remodeling of the extracellular matrix in the ER+ tumor environment, including structural modifications predicted to enhance metastasis, confirmed in a mouse model of ER+ tumors [154].

### 5.5. Summary

In summary, estrogen activity modulates the expression of many immunomodulators and modifiers of the extracellular matrix in multiple target cell types that are present in the TME of ER+ breast cancers, consistent with widespread estrogen receptor expression (Section 4, Figure 1). As shown in Figure 2, the changes in these mediators have the potential to tilt the activity of key cytokine/chemokine axes and enhance recruitment and/or functional activity of non-tumor cells in the TME. The responses of the ER+ tumor cells themselves extend these observations beyond application to other solid tumors, and underscore the consequences of exposure to estrogen in untreated ER+ breast cancers, and responses to therapeutic anti-estrogens. While many of these estrogen-induced changes can be interpreted as “pro-tumor”, some activities, such as reduction of TGFβ and CCL2-induced recruitment of NK cells, would not. Functional outcomes would depend on the sum of systemic and local signals. As discussed in Section 6 below, analyses of experimental and clinical ER+ cancers are beginning to reveal these net effects over time; increased application of single-cell sequencing will illuminate the critical target cells and ligand/receptor networks [236].

The importance of studying primary tumor tissue is increasingly recognized, but this remains logistically difficult, and estrogen actions on individual cell types in the context of ER+ breast cancer have received little attention. MCF7 cells, including the studies cited above, represent much of what we know about direct estrogen signaling in ER+ breast cancer. However, despite their clear utility, these cells do not represent the diversity of clinical primary and metastatic ER+ breast cancers in women. Further, the response of ER+ tumor cells to estrogenic ligands will evolve as they accumulate somatic mutations and changes in their epigenomes, and respond to environmental cues. As discussed in Section 4, other signaling pathways, particularly NF-kB, and the specific estrogenic ligand can alter responses [167,170,172,237]. Stromal cells in the TME also display a spectrum of functional states that may shape their responses to estrogen activity, and result in significantly different responses from the model cell lines discussed here. Nonetheless, understanding the potential for estrogen regulation of components of cytokine networks and the extracellular matrix in the individual cell types illustrates candidate pathways that may underlie responses to estrogen agonists and anti-estrogens in ER+ breast cancer, and are worthy of additional study.

## 6. Net Outcomes of Manipulation of Estrogen Activity In Vivo

### 6.1. Effects of Manipulation of Estrogen Activity in Experimental Models

Multiple studies in defined in vitro experimental systems have demonstrated effects of manipulation of estrogen activity on the distinct cell types that populate the environments of ER+ breast cancers (reviewed in [23,24,30,31,32]), and the cytokine networks that regulate their expansion, recruitment, and functional activity, as described in Section 5 (Figure 2). However, the simplicity that facilitates these studies also limits interrogation of the dynamic interactions and functional plasticity of these cell populations in the in vivo environment. Experimental in vivo studies in models of cancers with genetic or pharmacological manipulation of specific estrogen receptors have demonstrated that estrogen/anti-estrogen activity can exert meaningful net effects on the stromal cells in vivo (e.g., [42,155]). As discussed in Section 5, ER+ cancer cells, such as breast cancer, can respond to estrogen activity with changes in expression of genes regulating the immune microenvironment and modification of the extracellular matrix (Figure 2A). The integrated impact of estrogen and anti-estrogen therapies at multiple targets on tumor behavior and disease progression is only beginning to be addressed in experimental models. Animal models enable interrogation of not only net outcomes, but also the roles of individual mediators and cell populations in orchestration of the response. Until mice with fully humanized immune systems become available, syngeneic models are required to reveal this interplay. In an elegant study of estrogen action in cancers that do not express estrogen receptors (ovarian, lung, and mammary ER-negative tumor models), Conejo-Garcia and his colleagues demonstrated that 17β-estradiol drove tumor growth by ERα-mediated expansion, mobilization, and activation of MDSCs (mostly gMDSCs). Although estrogen also measurably reduced the activity of cytolytic CD8+ and CD4+ T cells, the primary mediators of this effect were MDSCs [42]. Pietras and colleagues extended these observations using the 4T1 TNBC syngeneic mouse model, and observed that a novel SERD reduced intratumoral MDSCs and Tregs, and permitted increased CD8+ cell infiltration [160].

Del Rio Hernandez and colleagues demonstrated the functional importance of GPER, which can be activated by 17β-estradiol, as well as current SERMs and SERDS, in the desmoplastic response [155,193]. These changes in the composition and properties of the extracellular matrix drive proliferation, inflammation, and invasion of many cancers, including ER+ breast cancer. In mouse models of pancreatic ductal adenocarcinoma, tamoxifen reduced myofibroblastic differentiation, collagen fiber thickness and alignment, and transcripts for the crosslinking enzyme, LOXL2, fibrillar collagen, and other ECM proteins, such as FN1. This was associated with fewer macrophages exhibiting features of M2 polarization, reflecting the close relationship between features of the extracellular matrix and inflammation.

However, the paucity of immunocompetent models of ER+ breast cancer, which are required to reveal integration of signals through tumor ERα into the cytokine/chemokine network that regulates immune responses, feature limited studies of this disease. We have generated an immunocompetent mouse model of aggressive ER+ mammary cancer resulting from expression of transgenic prolactin in the mammary environment [238,239,240]. ER+ tumorigenesis is associated with accumulation of macrophages, but low lymphocytic infiltrate, and an immunosuppressed cytokine environment, including elevated TGFβ [240], resembling ER+ clinical disease. Supplementation with 17β-estradiol dramatically accelerates growth of the primary tumors and increases pulmonary metastatic burden [154]. Estrogen treatment remodeled the architecture of the peritumoral extracellular matrix, increasing transcripts for fibrillar collagens, remodeling enzymes, and matrisomal proteins which have been shown to contribute to increased alignment and stiffness [122,129,135]. This was associated with reorientation of collagen fibers more perpendicularly to the primary tumor boundary (TACS-3, [145]), an independent prognostic indicator of reduced disease-free survival for clinical ER+ breast cancer patients [144] and associated with elevated M2 macrophages [146]. Estrogen also increased POSTN and FN1 expression in the lung, which are linked to successful lung metastases [241,242], suggesting that estrogen can precondition the metastatic niche [154]. Furthermore, 17β-estradiol supplementation increased mRNAs for CXCL12, TGFβ1, and LTBP1, predicting heightened immunosuppression, metastasis, and cancer stem cell activity [211,215,216,217].

### 6.2. Effects of Anti-Estrogen Treatments on the Microenvironment of Clinical ER+ Cancers

Although studies of clinical disease are complicated by variability among patients, differing experimental designs and endpoints, these reports also highlight the crosstalk of inflammation with estrogen pathways (Section 4), and the multiple etiologies that may underlie anti-estrogen resistance [2,4,243]. As noted in Section 2, the frequency of TILs prior to treatment in ER+ breast cancers does not accurately predict prognosis, in contrast to other breast cancer subtypes [21,46,47,48,49]. Extension of these findings to evaluate total TILs as a potential predictor of response to anti-estrogen therapies e.g., Ki67, has yielded contradictory results (e.g., [48,244,245]).

Pre- and post-treatment transcriptional profiles of the heterogeneous ER+ tumor homogenate in response to AIs or fulvestrant in neoadjuvant studies of post-menopausal patients have enabled analysis of the sum of direct and indirect effects and compensatory responses after a relatively short (2–4 week) treatment course. Although the majority of these studies did not directly look at tumor-associated immune cells or the structure of the extracellular matrix, and post-treatment samples would be complicated by altered proportions of different cell populations, these studies demonstrate that reduction of estrogen activity by both ER degradation and reduced estrogen synthesis has dramatic effects on cytokine/chemokine networks, and components and modifiers of the extracellular matrix and tumor cell responses (such as TGFβ, PDGFRB, and the Hippo pathway) [246,247,248,249]. Intriguingly, longer-term (4–6 month) AI neoadjuvant treatment in two smaller studies showed a reduction in the frequency of FOXP3+ cells or the ratio of FOXP3+/CD8+ lymphocytes that was particularly strong in responders [250,251], consistent with estrogen-stimulated recruitment/differentiation of Treg CD4+ cells.

Other studies have demonstrated modulation of inflammatory signals by anti-estrogens. A recent study examined the effect of 3 weeks of exposure to neoadjuvant tamoxifen [172]. Although TIL frequency and the content of CD8+, CD4+ and CD68+ immune cell populations were not altered, tamoxifen selected for ER+ tumor cells driven by the inflammatory NF-κB pathway with elevated stem cell and epithelial mesenchymal transition features.

The converse, the importance of immune cell activity and interactions with the extracellular matrix in the response to reduced estrogenic activity, was shown in a recent study designed to identify features of ER+ cancers that predict de novo resistance to suppression of proliferation by AIs (luminal B cancers) [69]. Ellis and colleagues examined transcriptional profiles of ER+ tumors from postmenopausal patients from the Alliance and Preoperative Letrozole trials who failed to respond to 4 weeks of AI treatment. These luminal B cancers exhibited higher activity of pathways associated with the ECM-regulated Hippo pathway and immune tolerance, including mRNAs encoding checkpoint inhibitor molecules (IDO1, PD1, LAG3) and increased IFNγ-STAT1 signaling [69]. Immunocytochemical studies revealed increased IDO1 expression on macrophages in the stroma, as well as tumor cell nests, but these cells did not display M2 markers. This pattern was associated with elevated PD-1 and LAG3 expression on TILs, and increased FOXP3+ immunosuppressive Tregs. Interrogation of the TGCA and METABRIC databases confirmed that high expression of IDO1 and LAG3 was associated with poor survival in luminal B cancers.

Richer and her colleagues examined metastatic ER+ disease which exhibited acquired resistance to AIs secondary to mutant ESR1 (mESR1), resulting in constitutive activation of ERα [107]. Compared to metastatic lesions expressing wildtype ERα, tumors expressing mESR1 contained higher levels of FOXP3+ Tregs and CD4+FOXP3- lymphocytes, and macrophages expressing the checkpoint protein PD-L1, without altering numbers of CD8+ T cells and CD20+ B cells, consistent with heightened immunosuppression. Transcriptomic analyses of human breast cancer cells engineered to express mESR1 indicated enhanced activity of Type 1 IFN/STAT1 signaling and the innate immune pathway, demonstrating the importance of tumor autonomous activity. Findings in AI-treated ER+ cancers are complicated by the induced increase in circulating and intratumoral androgens [196], and some metastatic AI-resistant ER+ cancers with mESR1 exhibited increased AR expression/and target genes [107]. Nonetheless, these findings underscore the immune responses to standard-of-care manipulation of estrogen activity in treatment of ER+ disease.

### 6.3. Summary

Together, these reports confirm that estrogen activity can potently regulate immunomodulatory signals and the extracellular matrix which surrounds these cancers in vivo, across heterogeneous ER+ breast cancers. Some of the strongly influenced pathways are predicted by studies of individual cell populations, as discussed in Section 5; additional studies will be required to identify the target cells, functional status, and estrogen receptors which mediate these changes in vivo. Studies in experimental models, as well as analyses of patient tumors following neoadjuvant anti-estrogen therapies underscore the importance of the abundance and plastic functional status of macrophage and other myeloid immune subpopulations, as well as Treg lymphocytes in these cancers (Section 2). Moreover, the ability of anti-estrogen treatments to alter ECM components and remodeling enzymes supports the dynamic role that the extracellular matrix plays in these tumors, and the extensive interplay between the ECM and the immune environment, particularly macrophage activity.

## 7. Conclusions and Important Areas of Future Study

ERα marks heterogeneous clinical breast cancers which display a repertoire of somatic genomic mutations and immune environments that differ from other breast cancer subtypes. These cancers also exhibit distinct biological behaviors; despite an overall better prognosis than HER2+ or TNBC, disseminated dormant cells can lead to disease recurrence decades after the initial diagnosis and treatment. Estrogen exposure is linked to development of these cancers [252], and estrogen activity is a major driver of proliferation of most established tumors. Anti-estrogens with various modes of action constitute the primary standard-of-care adjuvant/neoadjuvant treatment and prevention strategies. After cessation of treatment, rising estrogen activity may contribute to re-activation of dormant cancers, as suggested by some preclinical xenograft studies [253], and reports of post-menopausal estrogen alone or estrogen/progesterone therapies on risk of recurrence [254,255,256,257].

It has long been recognized for many malignancies that a community of cell types and local communication networks support preneoplastic, primary, metastatic, and therapy-resistant lesions. A rich literature documents expression of diverse estrogen receptors on these non-tumor-cell populations, which is particularly relevant to the anti-estrogen therapies employed in ER+ disease, but also may play a role in other tumors. In contrast to other breast cancer subtypes, ERα+ breast cancers are characterized by direct actions of estrogenic ligands on cancer parenchyma. Among the estrogen- regulated genes in ERα+ cancer cells are many modifiers of immune recruitment/activity and the surrounding extracellular matrix, which would contribute to the orchestration of this network.

These interactions are dynamic. As disease progresses, cancer cells themselves are selected and/or epigenetically modified to adapt to metastatic sites, and the spectrum of associated stromal cell subpopulations functionally respond. Treatments, including anti-estrogens with different modes of action, exert distinct effects on many cell types in the microenvironment, depending on the agent, estrogen receptor repertoire, and specific cell context. An intricate chemokine network choreographs pro- and anti-tumor immunity by altering expression of ligands and/or receptors on immune cells, fibroblasts, and tumor cells (reviewed in [86]). Inflammation can promote carcinogenesis, or support anti-tumor immunity. Responses to even 17β-estradiol depend on growth factor/cytokine milieu [161,164,166], and estrogen can be pro- or anti-inflammatory [23,24,217]. Growing evidence supports the importance of crosstalk of estrogenic signals with important players. Estrogen and NF-κB can co-repress the other’s signals, or synergize to promote disease progression [167,172,237]. Similarly, the consequences of estrogenic regulation of the TGFβ pathway differ with disease stage [258,259]. Thus, tumor context is critical for understanding consequences of the different anti-estrogens on the cellular and biochemical signaling components in the microenvironment, and implications for patient responses. Ongoing development of proteolysis-targeting chimeras (PROTACs), which trigger degradation of mutant as well as wildtype ERα and next generation SERDs [260,261], will continue to improve efficacious inhibition of ERα-mediated signals.

Appreciation of the relationships among heterogeneous ER+ cancer cells and associated stromal cells, including not only macrophages of various polarization states, but also other cells of the myeloid lineage, CD8+ and Treg lymphocytes, CAFs and vascular endothelium, is critical to understanding ER+ disease, and how these relationships evolve with tumor progression, disease site, and therapies. The array of functional states and spatial relationships among these cells remains relatively unexplored [44,121]. However, the growing availability of technologies that permit spatial resolution and localize gene expression, and increasing incorporation of single cell sequencing analyses, will build our understanding of the major axes which fuel immunosuppression in ER+ cancers, and illuminate the contributions of estrogen activity and impact of therapeutic interventions. Such studies will provide the foundation to develop effective immune approaches for ER+ breast cancer.

Despite the low efficacy of immune checkpoint inhibitors in advanced ER+ disease, a small number of patients respond [50,51]. Recent reports of elevated expression of other checkpoint proteins (e.g., LAG3) in tumors with de novo resistance to AIs [69], and PD-L1 expressing-macrophages in metastases with acquired mutations in the ligand-binding domain of ESR1 [107] suggest subsets of patients who may benefit from this therapeutic strategy, and underscore the need to develop biomarkers to identify these individuals. Multiple ongoing trials are evaluating concomitant AIs or fulvestrant and immune checkpoint inhibitors (examples reviewed in [30,32]). These studies will also enable rigorous evaluation of the impact of the anti-estrogen-induced reduction in immunosuppressive MDSCs, predicted by preclinical studies [42]. Interestingly, co-targeting the epigenetic regulator, histone deacetylase, with tamoxifen and checkpoint inhibitors reduced the frequency of Tregs [262].

Beyond immune checkpoint inhibitors, enhanced understanding of the immune microenvironment of ER+ tumors, particularly at metastatic sites, will enable evaluation of extensive efforts in the cancer field to improve anti-tumor immune responses. Strategies to overcome the immunosuppressed microenvironment and increase CD8+ T cell infiltration, as for other immunologically “cold” cancers, are critical. Robust interest supports development of approaches to modulate functional states and/or manipulate recruitment of immune cells, such as macrophages, natural killer cells and γδ T cells, (reviewed in [61,72,94,124,150,263,264,265,266]). Beyond specific immune cell targets, modulation of other features and cell types in the ER+ TME such as the vasculature, CAFs, and properties of the extracellular matrix (reviewed in [124,149,150]), will address the interactive relationships among all of the components of the environment. Further, other approaches such as low-dose radiation of the primary tumor may be helpful to improve antigen presentation and modulate the immune environment to support a systemic anti-tumor response; development of molecularly targeted radionuclides may directly target disseminated disease [267].

Notably, anti-estrogen treatments are increasingly combined with other therapeutic modalities, such as CDK4/6 and PI3K/mTOR inhibitors. Like anti-estrogens, these agents act on multiple cell types beyond the ER+ tumor parenchyma, and they perturb multiple activities in addition to cancer cell proliferation and survival. For example, CDK4/6 inhibitors modulate immune functions, such as antigen presentation, and induce T cell memory in clinical and experimental melanomas [268,269]. Multiple clinical trials in ER+ breast cancer are evaluating treatment regimens which combine these approaches (reviewed in [32]). Initial studies are appropriately focused on patient outcomes, but appreciation of the differences among ER+ cancers and analyses of immune infiltrate/activity and cytokine expression will shed light on mechanisms and enable refinement of these strategies.

Preadjuvant trials in patients with ER+ breast cancer have provided invaluable insights into the integrated impacts of treatments in established primary tumors. Illumination of mechanisms would be facilitated by complementary experimental models. However, the paucity of immunocompetent models of ER+ breast cancer has limited study of the roles of individual cell types and mediators in modification of the local and systemic cytokine milieu, cancer cell invasion, colonization and outgrowth at distant sites, and assessment of the integrated sum of individual interventions on disease burden. The broad availability of genetic approaches and commercial reagents have made mice attractive experimental models for many cancers. However, immunocompetent murine models of ER+ breast cancer are rare. Our prolactin-induced spontaneous model of aggressive ER+ disease, the NRL-PRL mouse, is permitting interrogation of the mechanisms underlying development and progression of ER+ disease, and the role of estrogenic signals [154,238,239,240]. Ongoing refinement of humanized mouse models will enable study of PDX models of diverse ER+ breast cancers and interactions with their surrounding microenvironment, and development of efficacious targeted therapeutic approaches.

Metastatic ER+ breast cancer is a deadly disease. Appreciation of the genomic evolution of these cancer cells and development of targeted therapies has reduced mortality, yet growing recognition of the importance of the TME has not translated into new approaches to ER+ disease. Knowledge of the dynamic microenvironments of diverse ER+ breast cancers will inform our understanding of mobilization, metastatic colonization and disease recurrence, and the role of estrogen/anti-estrogen signals at multiple target cells in the underlying processes. These insights will provide the foundation for developing new treatment and prevention strategies to eliminate mortality of this disease.

## Figures and Tables

**Table 1:** Summary of effects of estrogen ligands at distinct estrogen receptors.

Estrogen Activity Modulator	Activity at ERα	Activity at Mutant ERα (mESR1)	Activity at ERβ	Activity at GPER
17*β*-estradiol	agonist	no effect; constitutively active	agonist	agonist
SERMs, e.g., tamoxifen	competitive antagonist to partial agonist, depending on cell context	potential new antagonists under development	partial agonist	agonist
SERDS, e.g., fulvestrant	competitive antagonist and degrades	can degrade; lower affinity	reduced ability to degrade	agonist
Aromatase inhibitors	reduces endogenous E2	no effect; constitutively active	reduces endogenous E2	reduces endogenous E2

## Supplementary Materials

The following are available online at https://www.mdpi.com/article/10.3390/cancers13153725/s1, Table S1: Expression of key genes in response to estrogenic ligands in various models.
